# Sertraline May Improve Language Developmental Trajectory in Young Children with Fragile X Syndrome: A Retrospective Chart Review

**DOI:** 10.1155/2012/104317

**Published:** 2012-05-31

**Authors:** Tri Indah Winarni, Weerasak Chonchaiya, Evan Adams, Jacky Au, Yi Mu, Susan M. Rivera, Danh V. Nguyen, Randi J. Hagerman

**Affiliations:** ^1^Medical Investigation of Neurodevelopmental Disorders (MIND) Institute and Department of Pediatrics, University of California-Davis Medical Center, Sacramento, CA 95817, USA; ^2^Division of Human Genetic, Center for Biomedical Research, Faculty of Medicine, Diponegoro University, Central Java Semarang 50231, Indonesia; ^3^Faculty of Medicine, Chulalongkorn University, Bangkok 10330, Thailand; ^4^Division of Biostatistics, Department of Public Health Sciences, University of California, Davis, CA 95817, USA; ^5^Department of Psychology, University of California, Davis, CA 95817, USA; ^6^Center for Mind and Brain, University of California, Davis, CA 95817, USA

## Abstract

Young children with fragile X syndrome (FXS) often experience anxiety, irritability, and hyperactivity related to sensory hyperarousal. However, there are no medication recommendations with documented efficacy for children under 5 years old of age with FXS. We examined data through a chart review for 45 children with FXS, 12–50 months old, using the Mullen Scales of Early Learning (MSEL) for baseline and longitudinal assessments. All children had clinical level of anxiety, language delays based on MSEL scores, and similar early learning composite (ELC) scores at their first visit to our clinic. Incidence of autism spectrum disorder (ASD) was similar in both groups. There were 11 children who were treated with sertraline, and these patients were retrospectively compared to 34 children who were not treated with sertraline by chart review. The baseline assessments were done at ages ranging from 18 to 44 months (mean 26.9, SD 7.99) and from 12 to 50 months (mean 29.94, SD 8.64) for treated and not treated groups, respectively. Mean rate of improvement in both expressive and receptive language development was significantly higher in the group who was treated with sertraline (*P* < 0.0001 and *P* = 0.0071, resp.). This data supports the need for a controlled trial of sertraline treatment in young children with FXS.

## 1. Introduction

Fragile X syndrome (FXS) is a single gene disorder caused by mutation in the fragile X mental retardation 1 (*FMR1*) gene located at Xq27.3. The full mutation of CGG repeat expansion (>200 repeats) in the 5′ untranslated region (UTR) region leads to transcriptional silencing of the gene and a lack of fragile X mental retardation protein (FMRP) resulting in FXS [1]. FXS is the most common inherited form of intellectual impairment known, and it is characterized by a broad spectrum of cognitive, behavioral, and emotional impairment. The level of cognitive impairment ranges from borderline to severe intellectual disability (ID), and it correlates with the level of FMRP in blood [2, 3]. The full mutation allele frequency of FXS is about 1 in 4,000 in the general population [4, 5]. 

FMRP, an RNA binding, stabilizing, and transporter protein, is essential for synaptogenesis and the maturation and pruning processes of dendrite spines during development and throughout life [6–8]. FMRP is also a regulator of translation, typically through suppression, so the lack of FMRP leads to excessive synthesis of proteins [9] and synaptic dysfunction throughout the brain [10]. FMRP is functionally linked to perhaps hundreds of mRNAs [11], so that its absence disrupts the neurochemical foundation of learning, memories, and behavior [12]. 

Behavioral and emotional impairment in FXS includes shyness, social avoidance, anxiety, tactile defensiveness, mood instability, irritability, impulsiveness, hyperactivity, aggression, self-injurious behavior, autism spectrum disorders (ASD), and aggression [13–18]. Many of these behaviors interfere with social interaction thereby further impacting language and learning [19]. Language development has a significant impact on overall cognitive abilities in FXS [20] and is also a critical domain to predict comorbid autism in children with FXS [21–23]. Receptive language is relatively less affected than expressive language in young children with FXS [20]. Likewise, the degree of communication deficit has an impact on the level of anxiety for children with autistic disorders [24]. Approximately, 30% of individuals with FXS have autistic disorder and another 30% have pervasive developmental disorder not otherwise specified (PDD NOS) [25]. These categories will be jointly referred to ASD throughout this paper. Those with FXS and comorbid autism have been shown to have lower cognitive, adaptive, motor, and language abilities compared to those with FXS without autism [21, 26–29].

Selective serotonin reuptake inhibitors (SSRIs) have been widely used to treat anxiety, depression, and obsessive compulsive disorder (OCD). One such SSRI, sertraline, has been approved by the Food and Drug Administration (FDA) as a treatment for OCD in children (age 6–17 years old). Another SSRI, fluoxetine, has been approved by the FDA as an antidepressant treatment in children over 7 years old. Over the past two decades, SSRIs have been increasingly prescribed to children with ASD. In an open trial of fluoxetine, improvements were seen in social, communication, and cognitive domains in 129 children (2–8 years old) with autism [30]. In 1997, Steingard et al. published a case series of nine children with autism (6–12 years) treated with a low dose of sertraline (25–50 mg daily). Eighty-nine percent showed significant improvement in anxiety, irritability, and transition-induced behavioral deterioration [31]. By contrast, a controlled trial showed that another SSRI, citalopram, was not effective in children with autism aged 5–17 years old [32]. Although sertraline has been shown to have some beneficial effects in children with ASD with relatively few adverse effects [33], it is not currently FDA approved to treat ASD in children. 

Serotonin is known to enhance synaptic modulation and refinement [34]. During the period of peak synaptogenesis in early brain development (the first 5 years of life), there is evidence in children with ASD that brain synthesis of serotonin is reduced [35–37]. Serotonin can upregulate neurogenesis in the animal and human hippocampus [38–41]. A recent report of the use of fluoxetine, in the mouse model of Down syndrome demonstrated enhanced neurogenesis and restoration of the expression of 5-hydroxytriptamine 1A (5-HT1A) receptor when used after birth. In this study, the levels of brain-derived neurotropic factor (BDNF) were increased with enhancement in cognition [42]. This is the first paper of an SSRI-enhancing neurogenesis in early development completed with recovery of memory performance in an animal model of a neurodevelopmental disorder. Increased BDNF levels in the CNS can also have beneficial effects in FXS, including reversal of the dendritic spine abnormalities in FXS [43, 44]. The finding of an alteration of serotonin synthesis in children with ASD and the important role of serotonin in postnatal brain development and neurogenesis suggest the need for exploring the use of an SSRI in early childhood to reverse these deficits in those with ID or ASD [37]. 

In our clinical practice, we currently often use sertraline, an SSRI, to treat anxiety in young children with FXS and we hypothesize that this treatment may also help language development in these children. Therefore, we report here a chart review carried out retrospectively, comparing young children with FXS treated with sertraline compared to those not treated with sertraline who were age matched with a similar baseline developmental level. We compared the developmental language testing that was carried out in the past in both groups.

## 2. Method

### 2.1. Subjects/Participants

We conducted an observational retrospective analysis of the longitudinal changes in the Mullen Scales of Early Learning (MSEL) scores over time in 45 young children with FXS aged 12–50 months (42 male, 3 female), seen between the years of 2004 and 2011. Participants were children diagnosed with FXS and seen both clinically and for research through a variety of studies including those diagnosed at the time of birth, those diagnosed through prenatal studies in a known carrier, and young children with developmental delay who were diagnosed with FXS. All families signed an informed consent for research studies that included genetic assessment of *FMR1* and for developmental testing in the past, although all were also followed clinically through the Fragile X treatment and Research Center at the MIND Institute at the University of California at Davis Medical Center. All children were confirmed to have the full mutation with or without mosaicism by molecular testing. Language delay and comorbid ASD were documented by the Autism Diagnostic Observation Schedule (ADOS) [45], the Autism Diagnostic Interview, Revised (ADI-R) [46], and the Diagnostic and Statistical Manual of mental disorders, fourth edition (DSM-IV) [47, 48]. In our chart review, we found 11 children who were assessed at baseline and then with followup assessments after sertraline treatment that was prescribed clinically to treat anxiety and social deficits, and another 34 children who were not taking sertraline and were similarly assessed over time. In our chart review, sertraline was administered as early as 18 months in this retrospective study. 

The control group represented children with FXS who were not treated with sertraline (OFF sertraline), who were matched on age, language delay, MSEL early learning composit (ELC), and ASD at baseline. There were a variety of reasons that the control group did not receive sertraline clinically: (1) they were seen at the MIND Institute before sertraline was recommended clinically at such a young age or (2) parental refusal, did not want to treat their children with medication at a young age or (3) adverse side effects and subsequent discontinuation within 1 month of treatment onset (*n* = 2). The treatment dose of sertraline ranged from 2.5 mg to 12.5 mg/day for at least a three-month period. Dosage typically began at 2.5 mg/day and was increased as tolerated (mean 5.85 mg/day, SD 2.51). Higher doses typically lead to hyperarousal, more tantrums, irritability, and/or aggression. Individuals in both groups received similar early interventions, that is, 1 or 2 times/week until preschool at which time daily special education was received including speech therapy and occupational therapy through their community during the followup time.  Table 1 summarizes age at baseline, time to first followup visit, and total length of followup by group. 

### 2.2. Instruments

The MSEL has been used to measure children's developmental status from birth to 69 months of age [49]. The MSEL includes the gross motor (GM), fine motor (FM), visual reception (VR), receptive language (RL), and expressive language (EL) domains to achieve a complete and differentiated view of development in young children. Age equivalent scores were generated from each domain, and an Early Learning Composite (ELC) standard score was computed based on raw scores of the five domains. 

ASD was diagnosed by standardized measures including the ADOS [45], the ADI-R [46], and the DSM-IV [47], followed by a multidisciplinary team consensus of ASD diagnosis, and this was documented in our charts [25]. 

### 2.3. Data Analysis

To assess the differential rate of change (improvement) in the MSEL, linear mixed effects models were used with group (ON or OFF sertraline as defined earlier), age/time of measurement (in months), and group by time interaction with primary outcomes as expressive and receptive language score. Here, we report results for raw scores (as results based on the corresponding age equivalent expressive and receptive scores were similar). We employed a significance level of 0.05, and analyses were performed using SAS version 9.2. Predicted expressive and receptive language scores based on the fitted mixed model are provided at 20 months (about baseline) and at 40 months (corresponding to ~20 months after baseline).

## 3. Results

We examined MSEL expressive and receptive language over time in 45 children whose age at baseline assessment ranged from 12 to 50 months of age and who had language delays and similar MSEL Early Learning Composite Scores (Table 2). This includes 11 children who received sertraline after a baseline assessment and 34 children who were not on sertraline throughout the duration of the observation (followup) period.

The incidence of ASD was similar in both groups (72.7%, or 8/11, and 79% or 27/34, resp.). The time from baseline to first followup for the ON sertraline group (mean 11.7 months, SD 7.0 months) was significantly shorter than that of OFF sertraline group (mean 19.4 months, SD 9.2 months, *P* = 0.0255). The total length of followup time for the ON sertraline group (mean 18.6, SD 8.61) was significantly shorter than that of OFF sertraline group (mean 24.7 months, SD 6.2 months, *P* = 0.039). For the 11 children in the ON sertraline group, five had one followup visit, three had three followup visits, and the remaining three each had four, five, and six followup visits. Among the 34 children who were not on sertraline, 12 had one followup visit, three had 3 followup visits, and two had 4 followup visits; 17 had only baseline measurements.

**Figure 1:**
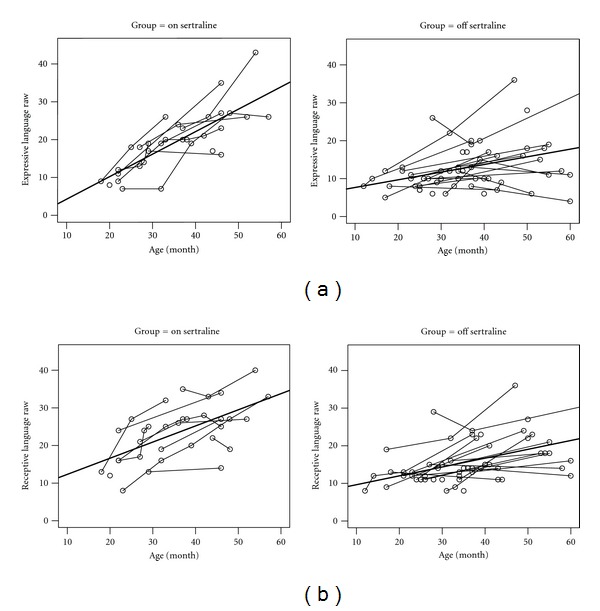
Expressive (a) and receptive language (b) trajectories over time for children on sertraline after baseline and children not on sertraline throughout the duration of the observation period.

As expected, language improvement was observed for all children over time (Figure 1). However, the rate of language improvement was significantly higher for children who were on sertraline after baseline compared to children who were not on sertraline through the observation period with respect to both expressive language (*P* < 0.0001) and receptive language (*P* = 0.0071). See Table 3 for details. For expressive language, the model-based mean MSEL score at age 20 months (about baseline) was similar for the ON sertraline group (mean 9.72, SE 1.89) and OFF sertraline group (mean 9.09, SE 1.20), but average scores at age 40 months between the groups were significantly different: mean 22.36 (SE 1.49) compared to mean 13.59 (SE 0.91), respectively. This similar pattern of improvement was also observed for receptive language (see Table 3 and Figure 1 for details).

## 4. Discussion

Assessment of the developmental trajectory in young children is very challenging, but it is necessary for a better understanding of developmental changes over time and can provide important information regarding effects of early treatment with sertraline in combination with early developmental interventions. From our retrospective chart review, we report here a better rate of improvement in language development over time for children with FXS who were treated with sertraline compared to children of similar ages and who did not receive sertraline treatment. Although this is an observational retrospective study which is not a treatment trial, the results suggest the need for a controlled trial of sertraline in young children with FXS. These data reflect our clinical experience with sertraline in treating young children with FXS, and we have seen improvements in anxiety, irritability, and socialization in addition to the language improvements noted in the MSEL assessments. 

Typically, the developmental trajectory in children with FXS is approximately 50% of the normal rate and expressive language is even lower [50]. A recent study reports that developmental delays in receptive and expressive language domains were evident by 9 month of age in children with FXS [51]. Language is an important domain because it most strongly correlates with intellectual ability. It is also the one of the main modalities through which a child relates to his environment, and it is an important conduit for social and emotional enrichment and stimulation from environment [20, 22]. Anxiety can interfere with social interaction, and it can also impair language development particularly in those with FXS [17, 24]. Early use of sertraline has been recently used clinically to improve both anxiety and social interaction in young children with FXS because of the emerging data regarding the use of an SSRI in young children with autism [30, 31, 36]. Because the clinical use of sertraline was not part of a study, we did not have followup measures of anxiety but instead we have only our routine MSEL testing that is carried out on all young children with FXS that we see clinically or for research. 

In our clinical experience, fluoxetine can often be too activating; however, sertraline in low dose (2.5 to 5 mg daily) is less activating and usually well tolerated in young children with FXS. Sertraline has minimal adverse effects compared to other SSRIs, and it also has minimal interference with the metabolism of other medications [52, 53].

 Of concern is the recent report regarding the use of citalopram (another SSRI) in autism [32]. This treatment did not demonstrate efficacy, but the age of the children treated was 5 to 17 years old [32]. Perhaps the effect that we see here in FXS is only apparent in young children under 5 years of age because this is a period of significant synaptogenesis. 

Sensory integration (SI) problems, characterized by inappropriate reactions to stimuli, have been reported in infants at 9–12 month of age with FXS [54]. At older ages, SI problems manifest as a variety of symptoms including tactile defensiveness, anxiety, hyperactivity, repetitive speech, hand flapping, rocking, and impulsivity [18, 29, 55–57]. We noted that treatment with sertraline often improved the anxiety and irritability of young children with FXS [31] and perhaps this improvement indirectly affected the language development of children with FXS. Alternatively, it is possible that sertraline may have a direct effect on the language areas of the brain through enhanced connectivity or neurogenesis.

It is essential for any pharmacological intervention to also be combined with early intervention from an educational standpoint. The effects of environmental stimulation in improving synaptic connections have been well demonstrated in FXS. Meredith and colleagues described that spike-timing-dependent long-term potentiation (STD LTP) in the prefrontal cortex, which is involved in higher cognitive function, was restored to wild-type (WT) level by an environmental enrichment in the *Fmr1* KO mouse [58]. In another study of the *FMR1*-KO mouse, an enriched environment rescued the abnormalities of the dendritic spines [59]. Enriched environments have antidepressant-like activity in animals and stimulate neurogenesis in the hippocampus [60]. All of the children who took part in this study received early intervention including speech and language therapy, occupational therapy (OT), and/or physical therapy (PT). However, the study here suggests that the use of sertraline can have a further beneficial effect at least with the trajectory of language development compared to those not treated with sertraline. 

The use of targeted treatments that will reverse the neurobiological consequences of the FMRP deficit such as metabotropic glutamate receptor 5 (mGluR5) antagonists and gamma-aminobutyric acid (GABA) agonists will likely be helpful for young children in the future, but these medications are not currently available to prescribe to young children [48, 61]. Sertraline is currently available, and further studies including a controlled trial are warranted in young children with FXS. 

## 5. Study Limitations

This is an analysis of retrospective data obtained in a chart review of medical records, and there are significant limitations in such a study. There can be clinical bias in who receives the sertraline and who does not in addition to other potential baseline confounders associated with receipt of treatment and with language development that were not available in the medical records. This analysis was based on observation data where the administration of sertraline was not as uniform, compared to a clinical trial study. Thus, there was variation in the start of sertraline after baseline for the 11 subjects on sertraline. Therefore, the results reported here should be interpreted with some caution and studies, including randomized controlled trials with a large number of patients so that individual differences in developmental trajectories will be also controlled for are needed to determine the efficacy of sertraline in young children with FXS.

## Figures and Tables

**Table 1:** Participant age, total length of observation (followup) times in months, and time to first followup by study group (ON/OFF sertraline).

Variable (month)	Group	Number	Mean	SD	Min	Median	Max
Age in months at baseline among all subjects	ON sertraline	11	26.91	7.99	18.0	23	44
	OFF sertraline	34	29.94	8.64	12.0	29.5	50

Age at baseline among subjects with at least 1 follow-up	ON sertraline	11	26.91	7.99	18.0	23	44
	OFF sertraline	17	25.41	7.23	12.0	25	37

Time to first followup visit	ON sertraline	11	11.73	6.97	4.1	9	24
	OFF sertraline	17	19.41	9.15	2.0	22	34

Total length of followup time	ON sertraline	11	18.55	8.61	4.1	17	34
	OFF sertraline	17	24.65	6.24	14.0	24	37

**Table 2:** Participants baseline MSEL receptive, expressive languages, and early learning composite score.

Group		Receptive raw	Expressive raw	Receptive T score	Expressive T score	Receptive age equivalent (mo.)	Expressive age equivalent (mo.)	ELC
ON sertraline	Mean	14.4	11.9	20.2	20.1	10.9	10.2	50.0
	STD	5.5	4.5	4.9	5.6	5.1	4.6	14.2
OFF sertraline	Mean	13.9	11.5	20.7	20.6	12.1	10.8	50.4
	STD	4.6	5.0	4.1	4.4	4.8	5.6	10.4

MSEL: Mullen Scale of Early Learning; STD: standard deviation; ELC: early learning composite.

**Table 3:** Change in expressive and receptive language (raw) MSEL scores.

					Average score at age 20 months				Average score, age at 40 months			
					ON sertraline		OFF sertraline		ON sertraline		OFF sertraline	
Outcome	Variable	Coefficient	SE^1^	*P* value	Estimate^2^	SE	Estimate	SE	Estimate	SE	Estimate	SE
Expressive language	Intercept	4.594	1.9994	0.0265	9.72	1.89	9.09	1.20	22.36	1.49	13.59	0.91
	Group (sertraline)	−7.5049	3.7628	0.0525								
	Age	0.2249	0.04961	<.0001								
	Age × group	0.4067	0.0948	<.0001								

Receptive language	Intercept	6.4653	1.837	0.001	15.32	1.81	11.47	1.13	25.14	1.45	16.48	0.89
	Group (sertraline)	−0.9751	3.4644	0.7797								
	Age	0.2504	0.04488	<.0001								
	Age × group	0.2408	0.08528	0.0071								

^1^SE : etandard error.

^2^Model-based estimate of mean expressive/receptive language score.
